# Kv1.3 Channel Blockade Modulates the Effector Function of B Cells in Granulomatosis with Polyangiitis

**DOI:** 10.3389/fimmu.2017.01205

**Published:** 2017-09-26

**Authors:** Judith Land, Lucas L. Lintermans, Coen A. Stegeman, Ernesto J. Muñoz-Elías, Eric J. Tarcha, Shawn P. Iadonato, Peter Heeringa, Abraham Rutgers, Wayel H. Abdulahad

**Affiliations:** ^1^Department of Rheumatology and Clinical Immunology, University of Groningen, University Medical Center Groningen, Groningen, Netherlands; ^2^Department of Internal Medicine, Division of Nephrology, University of Groningen, University Medical Center Groningen, Groningen, Netherlands; ^3^Kineta Inc., Seattle, WA, United States; ^4^Department of Pathology and Medical Biology, University of Groningen, University Medical Center Groningen, Groningen, Netherlands

**Keywords:** granulomatosis and polyangiitis, B cells, Kv1.3 potassium channels, antineutrophil cytoplasmic antibody, cytokines

## Abstract

B cells are central to the pathogenesis of granulomatosis with polyangiitis (GPA), exhibiting both (auto)antibody-dependent and -independent properties. Class-switched memory B cells in particular are a major source of pathogenic autoantibodies. These cells are characterized by high expression levels of Kv1.3 potassium channels, which may offer therapeutic potential for Kv1.3 blockade. In this study, we investigated the effect of the highly potent Kv1.3 blocker ShK-186 on B cell properties in GPA *in vitro*. Circulating B cell subsets were determined from 33 GPA patients and 17 healthy controls (HCs). Peripheral blood mononuclear cells (PBMCs) from GPA patients, and HCs were stimulated *in vitro* in the presence and absence of ShK-186. The production of total and antineutrophil cytoplasmic antibodies targeting proteinase 3 (PR3-ANCA) IgG was analyzed by enzyme-linked immunosorbent assay and Phadia EliA, respectively. In addition, effects of ShK-186 on B cell proliferation and cytokine production were determined by flow cytometry. The frequency of circulating switched and unswitched memory B cells was decreased in GPA patients as compared to HC. ShK-186 suppressed the production of both total and PR3-ANCA IgG in stimulated PBMCs. A strong decrease in production of tumor necrosis factor alpha (TNFα), interleukin (IL)-2, and interferon gamma was observed upon ShK-186 treatment, while effects on IL-10 production were less pronounced. As such, ShK-186 modulated the TNFα/IL-10 ratio among B cells, resulting in a relative increase in the regulatory B cell pool. ShK-186 modulates the effector functions of B cells *in vitro* by decreasing autoantibody and pro-inflammatory cytokine production. Kv1.3 channel blockade may hold promise as a novel therapeutic strategy in GPA and other B cell-mediated autoimmune disorders.

## Introduction

Granulomatosis with polyangiitis (GPA) is a severe autoimmune form of necrotizing small vessel vasculitis characterized by the presence of antineutrophil cytoplasmic antibodies (ANCAs) (1, 2). GPA patients frequently present with circulating ANCAs directed against the neutrophil constituent proteinase 3 (PR3). Several observations suggest that antineutrophil cytoplasmic antibodies targeting proteinase 3 (PR3-ANCA) play an important role in the pathophysiology of GPA. For example, leukocytes activated by ANCA release mediators that can injure endothelial cells *in vitro* and activation of neutrophils by ANCA can stimulate the release of neutrophil extracellular traps that contain chromatin and proteins including PR3. As B cells are the progenitors of ANCA-producing plasma cells (3), targeting B cells is an interesting therapeutic option for GPA.

Currently, patients are usually treated with broadly acting immunosuppressives. This strategy consists of cyclophosphamide and corticosteroids for induction therapy, often followed by azathioprine or mycophenolate mofetil (MMF) as maintenance treatment (4). While the introduction of immunosuppressive treatment has significantly improved the survival of GPA patients, severe adverse events are common, such as high rates of infections, thromboembolic complications, and drug toxicity (5). This emphasizes the need for more specific and less toxic treatment regimens for GPA patients. More recently, the anti-CD20 monoclonal antibody rituximab has been approved for induction therapy in ANCA-associated vasculitis. Rituximab was found to be non-inferior to standard cyclophosphamide treatment for induction of remission (6, 7). However, it was not possible to indicate rituximab as a clearly safer alternative to cyclophosphamide, as adverse event rates were similar (8). Moreover, there is a risk of persistent severe hypogammaglobulinemia and associated infections after rituximab treatment, necessitating IgG replacement therapy (9). Rituximab indiscriminately depletes all B cells, which may not be ideal as it has become evident that antibody-independent functions of B cells are also important in GPA (10). Certain B cells can exert regulatory functions, for example, through production of the regulatory cytokine interleukin (IL)-10 (11, 12). Conversely, B cells can also produce a variety of effector cytokines (13). Therefore, selectively targeting pro-inflammatory B cells without impairing the regulatory function of B cells may be preferable to targeting all B cells. As class-switched memory B cells have a higher propensity to undergo plasma cell differentiation and are important in the amplification and maintenance of autoimmune responses (14), targeting these B cells may hold therapeutic promise for autoimmune diseases in general and for GPA patients in particular.

It has been demonstrated that class-switched memory B cells express a significantly higher number of voltage-gated Kv1.3 potassium channels compared to other B cell subsets. These Kv1.3 channels can serve as a therapeutic target for modulation of class-switched memory B cell function (15). Similar to T cells, B cells use the Kv1.3 channels to regulate Ca^2+^ signaling by controlling the membrane potential. Activation of these lymphocytes induces intracellular Ca^2+^ release from internal stores. Depletion of these intracellular Ca^2+^ stores results in an influx of extracellular Ca^2+^. The driving force for Ca^2+^ entry is maintained by a counterbalance of K^+^ efflux mediated by Kv1.3 channels. This mechanism sustains elevated cytosolic Ca^2+^ levels required for optimal lymphocyte activation (16, 17). A potent peptide inhibitor of Kv1.3 channels termed ShK-186 has been identified and investigated for its modulatory effects on T cells (18). Considering the high expression levels of Kv1.3 channels on switched memory B cells, we hypothesized that blockade of these channels would result in inhibition of B cell effector functions. Therefore, we investigated the effect of Kv1.3 channel blockade on B cells *in vitro*, by determining its effect on ANCA production, B cell proliferation, and production of pro- and anti-inflammatory cytokines in GPA patients and healthy controls (HCs).

## Materials and Methods

### Study Population

Thirty-three PR3-ANCA-positive GPA patients (Table 1) and 17 age- and sex-matched HCs (8 males, 9 females, mean age 55.6 years, range 44.0–74.1 years) were enrolled in this cross-sectional study. The diagnosis of GPA was established according to the definitions of the Chapel Hill Consensus Conference (19), and all patients fulfilled the classification criteria of the American College of Rheumatology (20). Only patients without clinical signs and symptoms of active vasculitis and considered to be in complete remission, as indicated by the Birmingham Vasculitis Activity Score of 0 (21), were included in this study. None of the patients and controls experienced an infection at the time of sampling.

**Table 1:** Clinical and laboratory characteristics of the granulomatosis with polyangiitis (GPA) patients at the time of blood sampling.

	GPA patients
Subjects, *n* (% male)	33 (45)
Age, mean (range)	57 (26–85)
Antineutrophil cytoplasmic antibodies targeting proteinase 3 (PR3-ANCA^a^), *n* (% positive)	28 (85)
PR3-ANCA titer, median (range)	1:80 (0 to >640)
eGFR ml/min × 1.73 m^2^, median (range)	65 (13–111)
Disease duration in years, median (range)	12.5 (2.8–31.3)
Number of previous relapses, median (range)	2 (0–10)
Non/maintenance immunosuppressive therapy,^b^ *n*	20/13

*^a^ANCA-positive titer ≥1:40, ANCA-negative ≤1:20*.

*^b^Maintenance immunosuppressive therapy: azathioprine, azathioprine + prednisolone, prednisolone, mycophenolate mofetil + prednisolone, or methotrexate*.

At the time of inclusion, three GPA patients received azathioprine, one patient received azathioprine in combination with prednisolone, five patients were treated with low dose prednisolone, three patients received low dose prednisolone in combination with MMF, and one patient was treated with methotrexate. All patients had previously received a combination of cyclophosphamide with prednisolone as remission induction therapy for at least 12 months prior to their inclusion in this study. One patient received additional treatment with rituximab 1 year before inclusion, and two patients 5 years before inclusion (B cells were reconstituted in all). All subjects provided written informed consent and the study was approved by the Medical Ethical Committee of the University Medical Center Groningen. The main clinical and laboratory data of the patients are listed in Table 1.

### Flow Cytometry Immunophenotyping for B Cells

EDTA blood samples from GPA patients and HCs were washed twice with PBS + 1% BSA to remove plasma proteins. Next, 100 µl of the cell suspension was stained with CD19-eFluor450, CD27-APC-eFluor780 (both eBioscience, San Diego, CA, USA), IgD-PE (BD Biosciences, Franklin Lakes, NJ, USA), or the corresponding isotype controls. After 15 min, cells were fixed and erythrocytes were lysed using FACS lysing solution. Samples were washed and measured using an LSR-II flow cytometer (BD Biosciences) and data were analyzed using Kaluza 1.2 flow analysis software (Beckman Coulter, Brea, CA, USA). B cells were divided based on their surface expression of IgD and CD27. Results are expressed as percentages of total CD19^+^ B cells.

### Induction and Measurement of Total and PR3-ANCA-Specific IgG

Cell culture and quantification of total and PR3-ANCA IgG was performed as previously described by our group (22). Briefly, peripheral blood mononuclear cells (PBMCs) were isolated from GPA patients, who produce ANCA upon *in vitro* induction (20), and stored in RPMI 1640 (Lonza, Basel, Switzerland) supplemented with 50 µg/mL gentamycin (GIBCO, Life Technologies, Grand Island, NY, USA), 10% fetal calf serum (FCS, Lonza), and 10% dimethyl sulfoxide (DMSO). Cryopreserved PBMCs were thawed, and cell suspensions were adjusted to 10^6^ cells/ml in RPMI + 10% FCS. Cells were seeded in 48-well plates (Corning, NY, USA) and stimulated with 3.2 µg/ml CpG-oligodeoxynucleotides (ODN) 2006 (Hycult Biotech, Uden, the Netherlands), 100 ng/ml B cell-activating factor (BAFF; PeproTech Inc., Rocky Hill, NJ, USA), and 100 ng/ml IL-21 (Immunotools, Friesoythe, Germany) at 37°C with 5% CO_2_, in the presence and absence of 1 nM ShK-186 (Kineta Inc., Seattle, WA, USA). After 12 days, the culture supernatants were harvested and levels of both total IgG and PR3-ANCA IgG were determined using in-house enzyme-linked immunosorbent assay and Phadia ImmunoCAP 250 analyzer with EliA PR3*^S^* (Thermo Fisher Scientific, Waltham, MA, USA), respectively. Levels of PR3-ANCA IgG are expressed as response units (RU) per milliliter.

### B Cell Proliferation Assay

Thawed PBMC from GPA patients and HCs were stained with 2.5 µg/ml carboxyfluorescein diacetate succinimidyl ester (CFSE; Invitrogen, Life Technologies). Cells were then cultured at a concentration of 10^6^ cells/ml in RPMI + 10% FCS and stimulated using 3.2 µg/ml CpG-ODN 2006, 100 ng/ml BAFF, and 100 ng/ml IL-21, in the presence and absence of 1 nM ShK-186. After 4 days of incubation, cells were harvested, washed, and labeled with CD19-eFluor450, CD22-APC (BD Biosciences) and propidium iodide (BD Biosciences). Samples were measured using an LSR-II flow cytometer, and data were analyzed with Kaluza 1.2 software. The CFSE staining intensity of unstimulated B cells was used to determine the percentage of proliferated B cells. Proliferated B cells are expressed as the percentage of B cells that have undergone at least one round of cell division.

### Cells Stimulation and Measurement of Intracellular B Cell Cytokines

Thawed PBMCs were seeded in 24-well flat bottom plates (Corning) at 10^6^ cells/ml in RPMI + 10% FCS. Cells were stimulated with 500 ng/ml CpG-ODN 2006 in the presence and absence of 1 nM ShK-186. Plates were incubated for 72 h at 37°C with 5% CO_2_. During the last 5 h of incubation, cells were restimulated with 50 ng/ml phorbol myristate acetate (PMA; Sigma-Aldrich, St. Louis, MO, USA) and 2 mM calcium ionophore (Sigma-Aldrich). As a negative control, one sample was kept without restimulation. To inhibit cytokine release from cells, 10 µg/ml brefeldin A (Sigma-Aldrich) was added to each sample. Subsequently, cells were harvested, washed with PBS + 5% FCS, and stained using CD19-eFluor450 and CD22-PeCy5 (Biolegend, San Diego, CA, USA). Next, cells were fixed, washed, permeabilized using an Invitrogen Fix&Perm kit, and stained with IL-10-PE (Biolegend), tumor necrosis factor alpha (TNFα)-Alexa Fluor 488 (BD Biosciences), IL-2-PeCy7 (eBioscience), and interferon gamma (IFNγ)-Alexa Fluor 700 (BD Biosciences). Samples were measured using an LSR-II flow cytometer, and data were analyzed with Kaluza 1.2. Samples that were not stimulated with PMA + calcium ionophore were used as negative controls in order to set gates during data analysis. Data are presented as the total percentage of cytokine-positive cells within the CD19^+^CD22^+^ B cell population.

### Statistical Analysis

Statistical analysis was performed using SPSS v22 (IBM Corporation, Chicago, IL, USA) and Graphpad Prism v5.0 (GraphPad Software, San Diego, CA, USA). Data are presented as median values with an interquartile range unless stated otherwise. Data were analyzed with the D’Agostino and Pearson omnibus normality test for Gaussian distribution. For comparison between groups, the unpaired *t*-test was used for data with Gaussian distribution and the Mann–Whitney *U* test for data without Gaussian distribution. For paired comparisons, the paired *t*-test or Wilcoxon matched pairs test were applied for Gaussian and non-Gaussian data, respectively. Correlation analysis was performed using the Spearman rank correlation coefficient. *p* Values <0.05 were considered statistically significant.

## Results

### Distribution of Circulating B Cell Subsets in GPA

**Figure 1:**
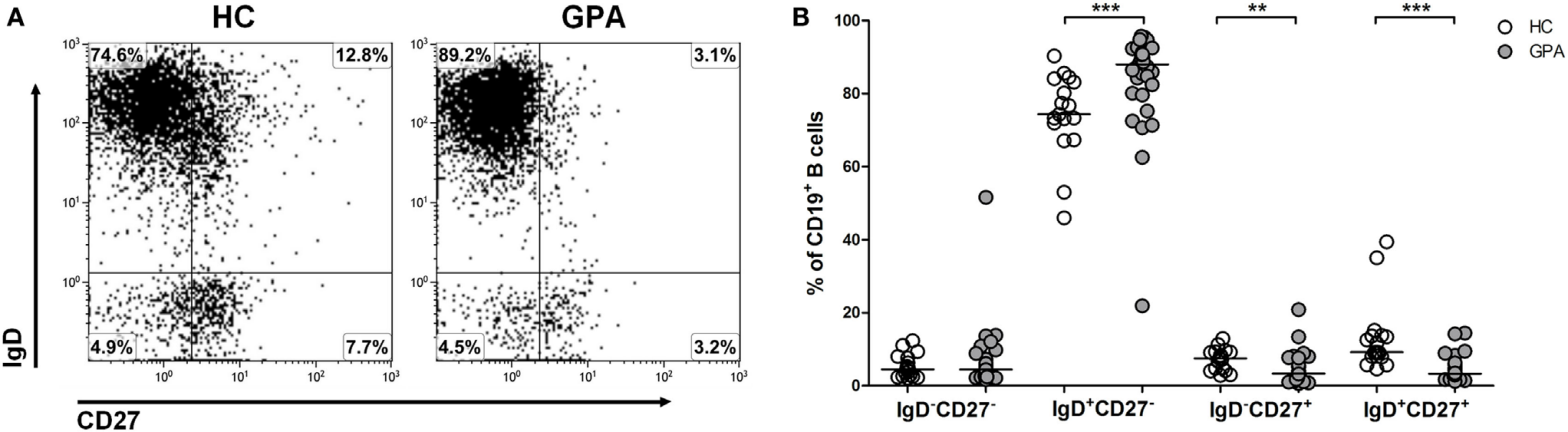
Phenotypic characterization of circulating B cell subsets in granulomatosis with polyangiitis (GPA) patients and healthy controls (HCs). **(A)** Flow cytometry gating strategy to distinguish differentiation subsets within peripheral blood CD19^+^ B cells. IgD^+^CD27^−^ transitional/naive B cells, IgD^+^CD27^+^ unswitched memory B cells, IgD^−^CD27^+^ switched memory B cells and IgD^−^CD27^−^ double negative B cells were identified. **(B)** Relative distribution of distinct CD19^+^ B cells subsets from HCs (open circles) and GPA patients (gray circles). Horizontal lines indicate median values. Graphs represent data of 17 HCs and 33 GPA patients (**p < 0.01, ***p < 0.001).

To evaluate the distribution of different B cell subsets, phenotypic characterization of circulating B cell populations was performed on blood samples from 33 GPA patients and 17 matched HC. B cells were identified using CD19 and surface expression of CD27 and IgD was used to distinguish four B cell subsets; IgD^+^CD27^−^ transitional/naive B cells, IgD^+^CD27^+^ unswitched memory B cells, IgD^−^CD27^+^ switched memory B cells, and IgD^−^CD27^−^ double negative B cells (Figure 1A). The percentage of double negative B cells did not differ between GPA patients (median 4.4, interquartile range 2.6–6.7%) and HC (4.5, 2.6–7.8%). An increased proportion of transitional/naive B cells was detected in GPA (88, 81–91%) compared to HC (74, 70–84%). Finally, GPA patients had lower percentages of both switched (3.4, 2.1–5.5% vs. 7.5, 4.5–9.3%) and unswitched memory B cells (3.3, 2.4–6.0% vs. 9.2, 8.0–14%) compared to HCs (Figure 1B).

### ShK-186 Inhibits PR3-ANCA IgG Production *In Vitro*

**Figure 2:**
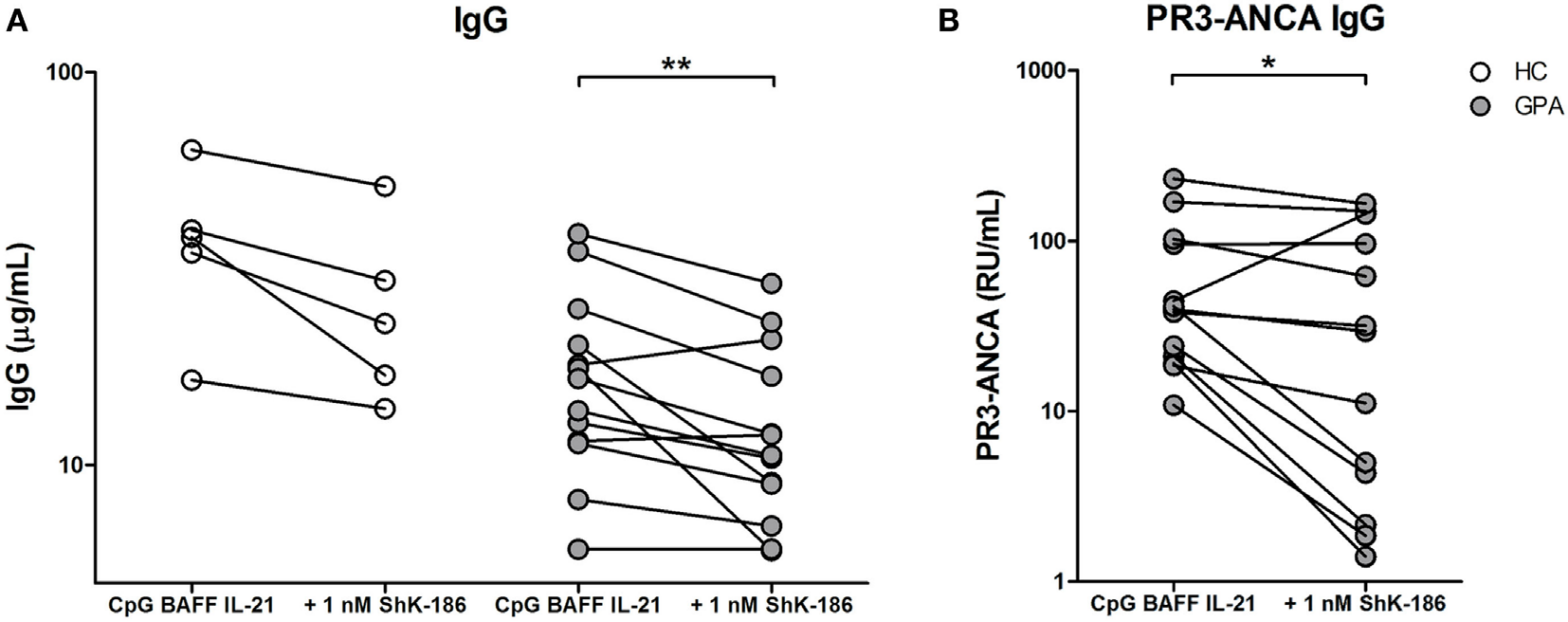
ShK-186 inhibits antineutrophil cytoplasmic antibodies targeting proteinase 3 (PR3-ANCA) production *in vitro*. **(A)** IgG production after peripheral blood mononuclear cell (PBMC) stimulation with CpG, B cell-activating factor (BAFF), and interleukin (IL)-21 from 5 healthy controls (HCs) (open circles) and 13 granulomatosis with polyangiitis (GPA) patients (gray circles) in the presence and absence of 1 nM ShK-186. **(B)** PR3-ANCA production after PBMC stimulation with CpG, BAFF, and IL-21 from 13 GPA patients in the presence and absence of 1 nM ShK-186 (*p < 0.05, **p < 0.01).

We next evaluated the effect of Kv1.3 channel blockade on the production of total and PR3-ANCA IgG by B cells *in vitro*. To investigate this, we performed our previously established method for inducing ANCA production *in vitro* by specific stimulation of B cells using CpG-ODN, IL-21, and BAFF (22). Only cells from GPA patients that produced ANCA *in vitro* were included in this study. PBMC from 13 GPA patients and 5 HCs were cultured in the presence and absence of ShK-186. Addition of 1 nM ShK-186 only at the start of the cell culture resulted in a significantly decreased production of total IgG (23, 15–40 µg/ml) compared to PBMCs cultured without ShK-186 (38, 26–52 µg/ml) in HC samples. IgG production from GPA patient samples (17, 11–23 µg/ml) was similarly reduced by treatment with 1 nM ShK-186 (11, 8–19 µg/ml) (Figure 2A). The effect of ShK-186 on IgG production was dose dependent (Figure S1A in Supplementary Material). Additionally, PR3-ANCA specific IgG production (40, 20–100 RU/ml) was significantly inhibited by ShK-186 (29, 3–120 RU/ml) in GPA samples (Figure 2B). Five patients showed a strong reduction in PR3-ANCA-specific IgG production by ShK-186. In seven patients, the effect of ShK-186 on the PR3-ANCA IgG was intermediate to limited, whereas inexplicable, one patient showed increased PR3-ANCA production after treatment with 1 nM ShK-186. Overall, the effect of ShK-186 appeared to be more pronounced on PR3-ANCA IgG production (median reduction of 39%) than on total IgG production (23%) in samples from GPA patients.

Production of total IgG in GPA correlated positively with the presence of IgD^−^CD27^+^ switched memory B cells (spearman’s rho = 0.62, *p* = 0.025), while production of PR3-ANCA-specific IgG did not (spearman’s rho = 0.03, *p* = 0.92), nor did production of PR3-ANCA IgG correlate with other B cell populations.

### ShK-186 Does Not Affect B Cell Proliferation

**Figure 3:**
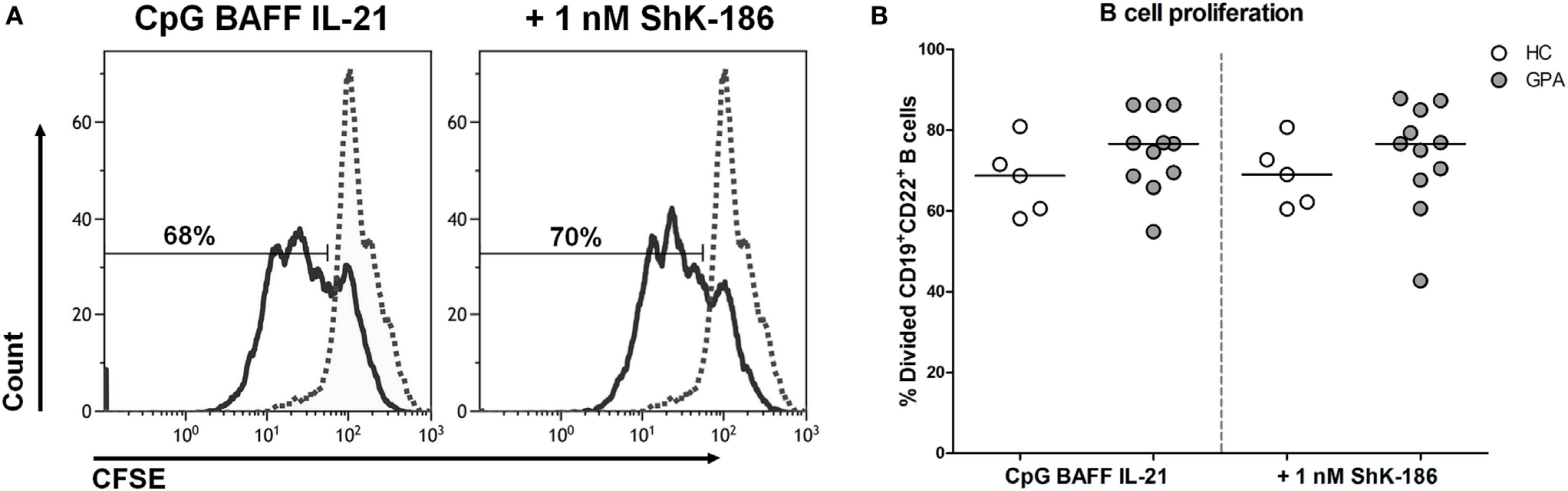
ShK-186 does not affect B cell proliferation. **(A)** Representative histograms of carboxyfluorescein diacetate succinimidyl ester (CFSE)-labeled B cells from one granulomatosis with polyangiitis (GPA) patient showing the effect of 96 h treatment with CpG, B cell-activating factor (BAFF), and interleukin (IL)-21 on B cell proliferation in the presence and absence of 1 nM ShK-186 (solid lines). Dashed lines represent unstimulated CFSE-labeled B cells. **(B)** Percentages of CD19^+^CD22^+^ B cells that have undergone at least one division after 96 h stimulation with CpG BAFF and IL-21 in the presence and absence of 1 nM ShK-186 from healthy controls (HCs) (open circles) and GPA patients (gray circles). Horizontal lines indicate median values. Graphs represent data of 5 HCs and 11 GPA patients.

To determine whether the decreased production of IgG and PR3-ANCA upon ShK-186 treatment occurs due to decreased B cell proliferation, the effect of ShK-186 on B cell proliferation was assessed in samples from 11 GPA patients and 5 HCs. As expected, stimulation of PBMC with CpG, BAFF, and IL-21 induced B cell proliferation (Figure 3), whereas no such proliferation was observed in other lymphocytes (data not shown). As shown in Figure 3B, proliferation of B cells from GPA patients was not suppressed by addition of 1 nM ShK-186. Similar results were obtained in HC samples without and with 1 nM ShK-186 (Figure 3B). There was no difference in the percentage of apoptotic B cells between cultures of GPA patient and HC samples with or without ShK-186 incubation (Figure S2 in Supplementary Material). These data indicate that the reduced IgG production upon ShK-186 treatment is not caused by decreased B cell proliferation.

### ShK-186 Suppresses Pro-inflammatory Cytokine Production by B Cells with Minor Effect on Anti-inflammatory IL-10 Expression

**Figure 4:**
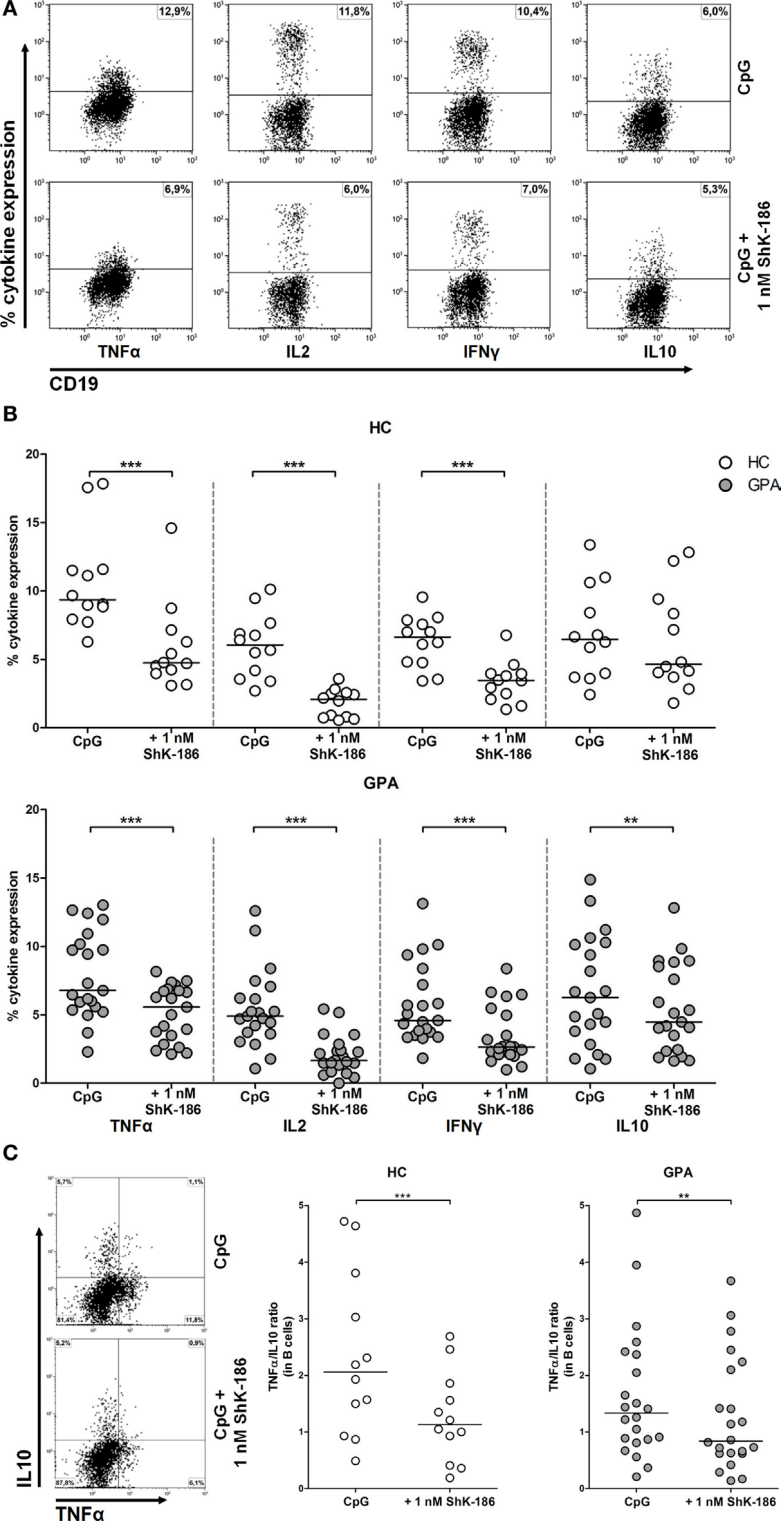
Effect of ShK-186 on intracellular cytokine production by circulating B cells. **(A)** Representative flow cytometry dot plots of cytokine production in CD19^+^CD22^+^ B cells from a granulomatosis with polyangiitis (GPA) patient. Upper panel represents cytokine production after CpG stimulation; lower panel represents cytokine production after CpG stimulation in the presence of 1 nM ShK-186. **(B)** Percentages of cytokine producing B cells after stimulation with CpG in the presence and absence of 1 nM ShK-186 from healthy controls (HCs) (upper panel) and GPA patients (lower panel). Horizontal lines indicate median values. Graphs represent data of 12 HCs and 21 GPA patients. **(C)** For all GPA patients and HC, the single-positive B cells for either tumor necrosis factor alpha (TNFα) or interleukin (IL)-10 were determined in the presence and absence of 1 nM ShK-186 (left panel) and the TNFα/IL-10 ratio was calculated (right panel) (**p < 0.01, ***p < 0.001).

In addition to antibody production, B cells can also participate in orchestrating the immune response by producing a wide range of pro- and anti-inflammatory cytokines. Therefore, we determined whether cytokine production by circulating effector B cells (TNFα, IFNγ, and IL-2) or regulatory B cells (IL-10) was influenced by blockade of the Kv1.3 channel (Figure 4A). Samples from 21 GPA patients and 12 HCs were stimulated in the presence and absence of ShK-186 and B cell cytokine production was assessed. As shown in Figure 4B, addition of ShK-186 to cell cultures significantly reduced the production of TNFα, IL-2, and IFNγ in samples from both HCs and GPA patients. The effect of ShK-186 on production of TNFα, IL-2, and IFNγ was dose dependent in both GPA and HC samples (Figure S1B in Supplementary Material). For IL-10, no significant effect of ShK-186 was observed in HC samples while in B cells from GPA patients a significant reduction was found (Figure 4B). Remarkably, the suppressive effect of ShK-186 on IL-10 production was less pronounced than that on TNFα, IL-2, and IFNγ.

Next, we assessed whether ShK-186 modulates the ratio of effector B cells to regulatory B cells. In this analysis, we defined effector B cells as TNFα^+^IL-10^−^ and regulatory B cells as TNFα^−^IL-10^+^ cells. The B effector:B regulatory ratio was significantly decreased after treatment with ShK-186 in GPA patients and HCs (Figure 4C).

Since activated T cells also upregulate Kv1.3 channels, one could argue that the observed effect on B cell cytokine production is due to the inhibitory effect of ShK-186 on activated T cells. To elucidate whether the beneficial effect of ShK-186 on B cells is T-cell dependent or independent, we performed a T-cell depletion experiment as described in Figure S3 in Supplementary Material. The results indicate that ShK-186 can modulate the production of B cell pro-inflammatory cytokines in the absence of T cells indicating that the effect of ShK-186 on B cell cytokine production is not caused by an indirect effect on T cells.

## Discussion

Granulomatosis with polyangiitis is a relatively rare but deadly disease when left untreated. Current treatment is unsatisfactory as it includes strong immunosuppressives with many severe side effects and is not always effective (23). Therefore, selective targeting of pathogenic pathways may hold therapeutic promise for GPA patients. Given the proposed pathogenic role of B cells and ANCA in GPA, total B cell depletion with rituximab seems a logical strategy for treatment. However, as a fraction of B cells exerts regulatory functions, it is important to identify a targeting strategy that can specifically inhibit effector functions of B cells, leaving suppressor aspects intact. Here, we evaluated the effect of blocking Kv1.3 channels, which are highly expressed by switched memory B cells, on (auto)antibody production, proliferation, and production of pro- and anti-inflammatory cytokines in B cells from GPA patients.

In the present study, we confirm previously described differences in the distribution of circulating B cell subset between GPA patients in remission and HC (22, 24). We observed a diminished percentage of circulating CD27^+^ memory B cells and an increased percentage of transitional/naïve B cells in GPA patients. A decrease in circulating memory B cells most likely represents an enhanced differentiation toward plasma cells (25, 26) or selective migration to inflammatory sites. B cell clusters have indeed been observed in granulomatous lesions of GPA patients (27). Therefore, we cannot exclude the possibility that the altered proportion of circulating B cell subsets in GPA patients occur due to migration of memory B cells toward inflamed tissue sites.

Differences in potassium channel expression on the surface of B cell subsets may contribute to changes in Ca^2+^ signaling patterns which influence the function of distinct B cell subsets as it does for T cell subsets (16, 17). Lymphocyte activation results in Ca^2+^ induced signaling pathways mediate *via* Kv1.3 channels. It has been demonstrated that the biophysical properties of Kv1.3 channels in human B cells parallels those in T cells (15). For instance, activation of switched memory B cells induces intracellular Ca^2+^ release from internal stores. Depletion of these Ca^2+^ stores causes calcium release-activated calcium channels to open the membrane, resulting in a Ca^2+^ influx. The driving force for Ca^2+^ entry is maintained by a counterbalance of K^+^ efflux mediated by Kv1.3 channels. This mechanism sustains elevated cytosolic Ca^2+^ levels required for optimal B cell activation affecting cellular processes such as (auto)antibody and cytokine production.

Blocking Kv1.3 channels on activated B cells *in vitro* inhibits the production of both total and PR3-ANCA-specific IgG. The mechanisms behind this effect are not fully understood. It has been shown that Kv1.3 channels on B cells are mainly expressed on IgD^−^CD27^+^ switched memory B cells (15), and total IgG production *in vitro* was indeed associated with this B cell subset. This association indicates that direct inhibition of switched memory B cells may explain the decreased production of total IgG. However, production of PR3-ANCA-specific IgG was not positively associated with the IgD^−^CD27^+^ B cell subset. The difference in the amount of autoreactive B cells present in the cultured samples could explain why IgD^−^CD27^+^ B cells not correlated with PR3-ANCA IgG. Interestingly, the production of total IgG was not completely inhibited by ShK-186. This might indicate that not all IgG-producing B cells were equally influenced by the Kv1.3-blocker, which can be considered as an advantage for this treatment. Consistent with our finding, Matheu and co-workers have demonstrated that the efficacy of ShK-186 is achieved without generalized immunosuppression *in vivo* (28). Proof-of-concept studies in rats showed a normal clearance of influenza virus and *Chlamydia* infection after treatment with ShK-186, whereas clearance of these infections was significantly delayed in rats treated with steroids (28). Thus, these studies might suggest that ShK-186 does not compromise the protective immune response against acute infectious agents.

Nevertheless, in the majority of patients, addition of ShK-186 did result in reduction of PR3-ANCA IgG production *in vitro*. A plausible explanation for the effect of ShK-186 on PR3-ANCA IgG production is that Kv1.3 channel blockade reduces the number of plasma cells formed *in vitro*, thus affecting the number of cells producing (PR3-ANCA) IgG. However, total B cell proliferation was not affected by ShK-186, suggesting that reduced proliferation is not the underlying cause for the lower IgG levels observed. It is also highly unlikely that the observed reduction in PR3-ANCA production is owing to the inhibitory effect of ShK-186 on activated memory T cells, as only B cells are activated by CpG *in vitro*. Therefore, the *in vitro* culture system present in this study provides a useful tool to investigate immunomodulation of (auto)antibody-producing B cells.

In contrast to our study, Wulff et al. previously reported an inhibitory effect of ShK on proliferation of B cells (15). They demonstrated that proliferation of IgD^−^ B cells (switched memory B cells) was suppressed in a dose-dependent manner, whereas proliferation of IgD^+^ B cells (including naïve and unswitched memory B cells) was inhibited only when using a high dose (100 nM) of ShK. Differences in methodology, stimulation conditions, and most likely B cell source, may be potential explanations for this apparent discrepancy between the studies. In their study, Wulff et al. used for their proliferation experiment human tonsillar CD19^+^ B cells and separated the B cell fraction into IgD^+^ cells (including naïve and unswitched memory B cells) and IgD^−^ cells (switched memory B cells). In our study, PBMCs were isolated from whole blood from patients and HCs. As shown in Figure 1, 88% of B cells from GPA patients and 74% of B cells from HC within the PBMCs fraction consist of transitional/naïve B cells, which according to the report by Wulff et al., are less prone to be suppressed in proliferation by ShK compared to IgD^+^ B cells. Therefore, in our study, the large proportion of naïve B cells within the total B cell population may have masked the suppressive effect of ShK-186 on switched memory B cells.

Production of cytokines by B cells was also clearly inhibited when samples were treated with ShK-186. This effect appeared to be more pronounced on the effector cytokines TNFα, IL-2, and IFNγ than on the regulatory cytokine IL-10. Indeed, when the TNFα/IL-10 ratio was determined, this significantly decreased upon treatment with ShK-186. The decrease in the TNFα/IL-10 ratio suggests that Kv1.3 channel blockade results in a relative increase of regulatory B cell phenotype compared to effector B cells. This may have positive effects in the treatment of patients with autoimmunity, as ShK-186 modulates the B cell response toward a suppressive phenotype.

The importance of targeting memory B cells is emphasized by the (lack of) efficacy of B cell-directed therapies. Treatment strategies affecting B cell survival, comprised of anti-BAFF with or without simultaneous blockade of a proliferation-inducing ligand (i.e., belimumab, tabalumab, and atacicept), have failed to show sufficient efficacy in clinical trials for treatment of systemic lupus erythematosus, rheumatoid arthritis (RA), and multiple sclerosis (29–34). Although these agents target mature B cells and short-lived plasma cells, the memory B cells are spared (35), and especially switched memory B cells appear to be resistant to BAFF depletion (36). By contrast, anti-CD20 B cell depleting studies using rituximab show beneficial outcomes for patients with GPA and RA (6, 37). However, disease flares have been observed in patients with RA when the memory B cell population returns following rituximab therapy (38). Thus, the current therapeutic agents do not adequately target memory B cells, but instead largely affect naïve B cells.

One could argue that only patients in remission, but not during active disease, have been included in this study. It has been suggested that upon active disease memory B cells migrate from the circulation into inflamed tissues of the upper airways or kidneys as a result of infectious triggers (25). Since migration of circulating memory B cells during active disease could alter the B cell subset distribution and exclude them from examination, this study was therefore focused on GPA patients during clinical remission. Furthermore, the stimulation conditions used *in vitro* in this study may mimic the stimulation by bacterial CpG motifs of autoreactive B cells inducing a pro-inflammatory milieu and ANCA production *in vivo*. However, studying patients with active disease would certainly be of interest.

## Conclusion

Considering the high expression of Kv1.3 channels on the surface of switched memory B cells and their involvement in autoimmune disorders, specific Kv1.3 channel blockade could be an attractive therapeutic target. Here, we demonstrate that ShK-186 is capable of inhibiting (auto)antibody and pro-inflammatory cytokine production *in vitro*, whereas production of the regulatory cytokine IL-10 was less affected. These results support the contention that selective targeting of Kv1.3 channels using ShK-186 may hold therapeutic promise for GPA.

## Ethics Statement

Written informed consent was obtained from all study participants. The study was approved by the institutional Medical Ethics Review Board of the University Medical Center Groningen (METc2012/151). All procedures were in accordance with the Declaration of Helsinki.

## Author Contributions

All authors contributed to the concept and design. JL and LL performed the experiments, statistical analysis, drafted the manuscript, and contributed to interpretation of the data. WA and PH contributed to interpretation of the data and critically revised the manuscript. AR and CS contributed to inclusion of patients with GPA, and assessed and participated in the interpretation of clinical data, and critical revision of the manuscript. EM-E, ET, and SI critically revised the manuscript. All authors read and approved the final manuscript.

## Conflict of Interest Statement

The authors declare that the research was conducted in the absence of any commercial or financial relationships that could be construed as a potential conflict of interest.

## Abbreviations

AAV, ANCA-associated vasculitis; APRIL, a proliferation-inducing ligand; BAFF, B cell-activating factor; BVAS, Birmingham Vasculitis Activity Score; CD, cluster of differentiation; ELISA, enzyme-linked immunosorbent assay; GPA, granulomatosis with polyangiitis; HC, healthy control; Ig, immunoglobulin; IFNγ, interferon gamma; IL, interleukin; MMF, mycophenolate mofetil; PR3-ANCA, antineutrophil cytoplasmic antibodies targeting proteinase 3; RA, rheumatoid arthritis; SLE, systemic lupus erythematous; TNFα, tumor necrosis factor alpha.
