# Supplementary material for: Kv1.3 Channel Blockade Modulates the Effector Function of B Cells in Granulomatosis with Polyangiitis
Source: Front Immunol. 2017 Sep 26;8:1205. doi: 10.3389/fimmu.2017.01205 (PMC5622953; doi:10.3389/fimmu.2017.01205)
Supplement: Supplementary file 1 [file Data_Sheet_1.PDF]

# Supplementary Material

# Kv1.3 channel blockade modulates the effector function of B cells in granulomatosis with polyangiitis.

Judith Land[1]§, Lucas L. Lintermans[1]§, Coen A. Stegeman[2], Ernesto J. Muñoz-Elías[3], Eric J. Tarcha[3], Shawn P. Iadonato[3], Peter Heeringa[4], Abraham Rutgers[1‡], Wayel H. Abdulahad[1,4‡*]

***Correspondence:** PhD. Wayel H. Abdulahad, w.abdulahad@umcg.nl

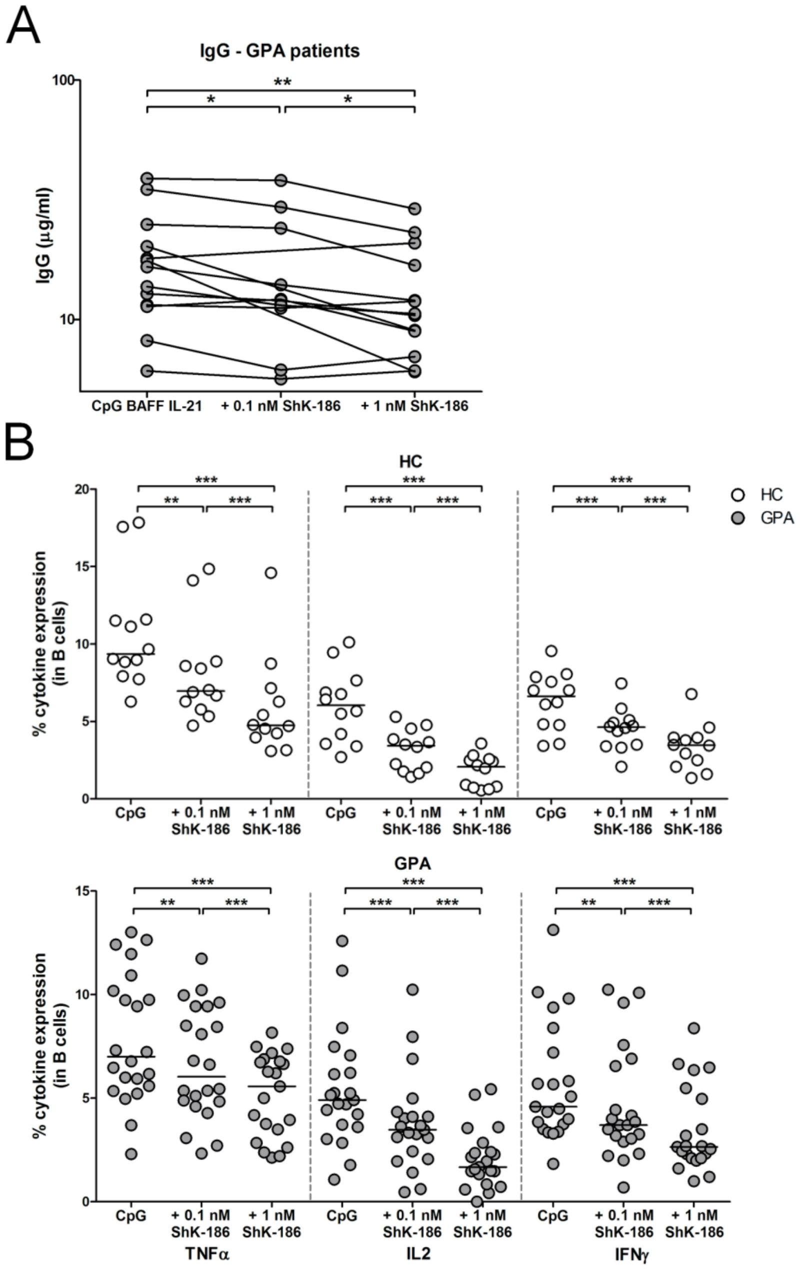


**Supplementary Figure 1: Dose-dependency A)** IgG production after PBMC stimulation with CpG, BAFF and IL-21 from 13 GPA patients in the presence and absence of 0.1 nM and 1 nM ShK-186. **B)** Percentages of B cells producing TNFα, IL-2 and IFNγ after stimulation with CpG in the presence and absence of 0.1 nM and 1 nM ShK-186 from HCs (upper panel) and GPA patients (lower panel). Horizontal lines indicate median values. Graphs represent data of 12 HCs and 21 GPA patients. *p<0.05, **p< 0.01, ***p< 0.001

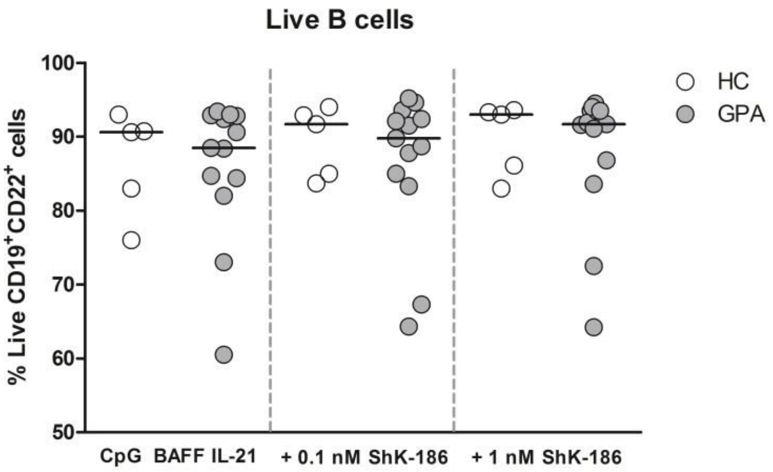


**Supplementary figure 2. ShK-186 does not affect B cell viability.** PBMCs from GPA patients and HCs were stimulated with CpG, BAFF, and IL-21 in the presence and absence of 0.1 and 1 nM ShK-186. After 4 days of incubation, cells were harvested, washed and labeled with CD19-eFluor450, CD22-APC and *propidium iodide (PI*). Samples were measured using an LSR-II flow cytometer and data were analyzed with Kaluza 1.2 software. The PI staining intensity B cells was used to determine the percentage of live B cells (i.e. PI negative $CD19^+CD22^+$ B cells). The graph represent the percentages of live $CD19^+CD22^+$ B cells from HCs (*n*=5, open circles) and GPA patients (*n*=11, grey circles) Horizontal lines indicate median values.

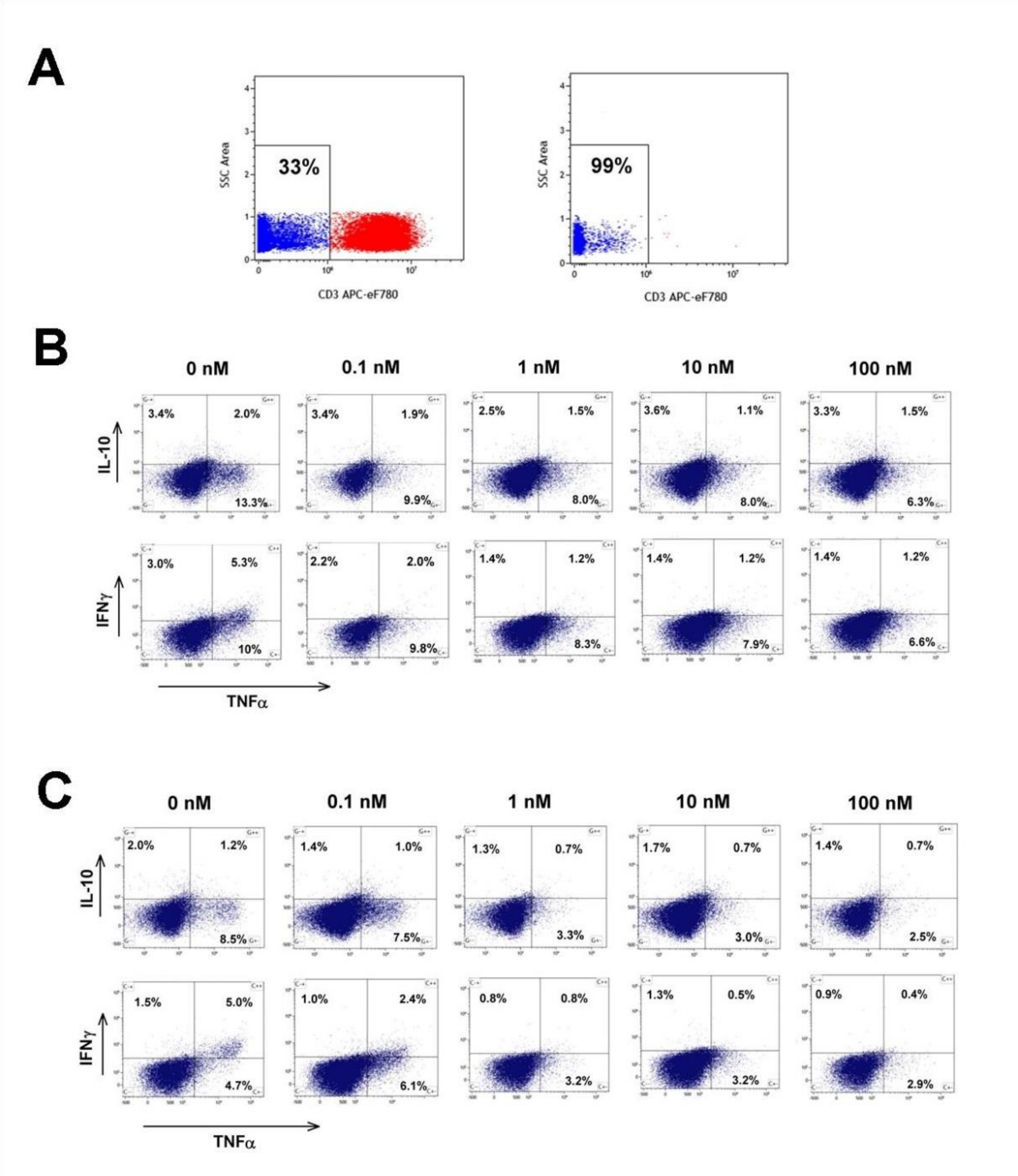


**Supplementary figure 3: Modulation of B cell pro-inflammatory cytokines by ShK-186 is T cell independent.**

PBMCs were isolated from 2 healthy volunteers and labeled with APC-eF780-conjugated anti-human CD3. Next, $CD3^+$ T-cells were depleted by FACS-sorting strategy. The purity of non-T cell fraction was 99% **(A)**. The non-T cell fraction was stimulated with CpG in the presence and absence of 4 different concentrations of ShK-186 (0.1, 1, 10, and 100 nM) and incubated for 72 hours at 37°C with 5% $CO_2$. During the last 5 hours of incubation cells were restimulated with 50 ng/ml PMA and 2mM calcium ionophore in the presence of Brefeldin A to inhibit cytokine release. Subsequently, cells were harvested, washed and stained using CD19-eFluor450 and CD22-APC. Next, cells were fixed, washed, permeabilized and stained with IL-10-PE, TNFα-Alexa Fluor 488, and IFNγ-BUV395. Samples were measured on LSR-II and data were analyzed with Kaluza 1.2. Dot plots of B cells from donor 1 (**B**) and donor 2 (**C**) expressing IL-10, TNFα, and IFNγ in the presence and absence of ShK-186.
